# The Role of Gut Microbiome in Psychiatric Disorders

**DOI:** 10.1192/j.eurpsy.2025.591

**Published:** 2025-08-26

**Authors:** J. T. Coelho, A. L. Ramos, B. C. da Silva, S. Timóteo

**Affiliations:** 1 São João Local Health Unit; 2 Santo António Local Health Unit, Porto, Portugal

## Abstract

**Introduction:**

Emerging evidence on the bidirectional connection between gastrointestinal microbiota and brain, through the gut-brain axis, and its influence on mental disorders makes the gut microbiota a potential target for novel therapeutic approaches.

**Objectives:**

We aim to study and synthetize the current data about the influence of gut microbiome on psychiatric disorders.

**Methods:**

Our literature research focused on some of the most significative English-written articles published in the last decade.

**Results:**

Most of the relevant literature suggests that the presence of a healthy and diverse gut microbiota is essential to normal cognitive and emotional processing. Also, it has been shown that consumption of probiotics can modify the functional activity of the areas in the brain that are implicated in cognitive functions.

The literature also supports that stress can change gut permeability as well as the composition of gut microbiota resulting in a pro-inflammatory profile of cytokines produced by gut microbiota. Besides, gut microbes can modulate the stress response and the level of anxiety through alterations in serotonin signaling.

It has been also demonstrated that in animal models of depression the composition of gut microbiota was changed. On the other hand, other studies demonstrated certain probiotics can attenuate depressive symptoms in rodent models.

Regarding eating disorders, Anorexia Nervosa seems to have impact on the gut microbiota balance through restrictive diets and the abrupt change in diet during nutritional rehabilitation. The use of prebiotics, probiotics, antibiotics or faecal transplantation looks promising as important novel adjuvant treatments.

**Conclusions:**

The effect of gut microbiota on several mental disorders is supported by a increased volume of experimental data.

However, research in this field is still unfolding and more studies should be performed to apply new techniques focusing on gut-brain axis in clinical practice.

**Disclosure of Interest:**

None Declared

